# Dissection of the regulatory role for the N-terminal domain in *Candida albicans* protein phosphatase Z1

**DOI:** 10.1371/journal.pone.0211426

**Published:** 2019-02-01

**Authors:** Krisztina Szabó, Zoltán Kónya, Ferenc Erdődi, Ilona Farkas, Viktor Dombrádi

**Affiliations:** Department of Medical Chemistry, Faculty of Medicine, University of Debrecen, Debrecen, Hungary; Louisiana State University, UNITED STATES

## Abstract

The novel type, fungus specific protein phosphatase Z1 of the opportunistic pathogen, *Candida albicans* (CaPpz1) has several important physiological roles. It consists of a conserved C-terminal catalytic domain and a variable, intrinsically disordered, N-terminal regulatory domain. To test the function of these domains we modified the structure of CaPpz1 by *in vitro* mutagenesis. The two main domains were separated, four potential protein binding regions were deleted, and the myristoylation site as well as the active site of the enzyme was crippled by point mutations G2A and R262L, respectively. The *in vitro* phosphatase activity assay of the bacterially expressed recombinant proteins indicated that the N-terminal domain was inactive, while the C-terminal domain became highly active against myosin light chain substrate. The deletion of the N-terminal 1–16 amino acids and the G2A mutation significantly decreased the specific activity of the enzyme. Complementation of the *ppz1 Saccharomyces cerevisiae* deletion mutant strain with the different CaPpz1 forms demonstrated that the scission of the main domains, the two point mutations and the N-terminal 1–16 deletion rendered the phosphatase incompetent in the *in vivo* assays of LiCl tolerance and caffeine sensitivity. Thus our results confirmed the functional role of the N-terminal domain and highlighted the significance of the very N-terminal part of the protein in the regulation of CaPpz1.

## Introduction

*Candida albicans* is an important opportunistic human pathogen that persists in the microbiome of healthy individuals causing slight candidiasis with hardly any serious symptoms under normal conditions [1]. On the other hand, in immune compromised patients, like the ones suffering from HIV infection or from autoimmune diseases as well as in immune suppressed recipients of organ transplants, the same fungal species can cause life threatening invasive infection. Additional predisposing diseases and conditions like cancer, diabetes, pregnancy or old age as well as steroid or antibiotics treatments of patients increase the prevalence of candidaemia. In fact *C*. *albicans* is one of the most common causes of nosocomial infections that can be propagated during hospitalization by catheters and other medical appliances [2–4]. The mortality rate associated with systemic bloodstream *C*. *albicans* infections is rather high, thus this fungus poses a considerable health hazard especially in developed countries [5]. At the moment echinocandins (anidulafungin, caspofungin, micafungin) and fluconazole are the gold standard in antifungal therapy; however, an increasing number of *C*. *albicans* strains are becoming resistant against these drugs [6–9]. Thus a search for new potential antifungal drug targets is well warranted, and a cellular signaling pathway directed approach seems to be a feasible strategy that one could follow [10].

Protein phosphorylation and dephosphorylation together constitute a general and fundamental regulatory cycle that controls nearly all of the vital functions in a eukaryotic cell, including signal transduction among others [11]. In this dynamic process protein kinases and phosphatases play an equally important role, thus the modulation of either of them can lead to the desired outcome in cellular response [12,13]. Since protein phosphatases are more divergent and less numerous than the kinases—that belong to a single enzyme family [14,15]—it is feasible to target the former especially if we can select a specific member of the family. Protein phosphatase Z (PPZ) is a “novel” type of Ser/Thr protein phosphatases that was first identified and characterized in *Saccharomyces cerevisiae* [16–17]. Its orthologs have a strictly restricted evolutionary distribution, they can be found only in the kingdom of fungi.

*Candida albicans* protein phosphatase Z1 was cloned and termed CaPpz1 [18]. It was shown to function in cation homeostasis, cell wall biosynthesis, and infectiveness [19], the regulation of hyphal growth [20], and in the oxidative stress response [21]. Thus CaPpz1 can be considered as a possible antifungal drug target as is fungus specific and has important physiological roles.

For a rational drug design project it is imperative to determine the structure and identify the possible regulatory mechanisms for CaPpz1. According to the published data, all of the known PPZ phosphatases consist of a conserved C-terminal catalytic domain and a highly variable intrinsically disordered (IDP) N-terminal domain [17,19]. The three dimensional structure of the C-terminal domain for CaPpz1 has been recently resolved by X-ray crystallography [22]. In agreement with the sequence based predictions [19], the overall structure of the CaPpz1 catalytic domain is similar to the catalytic subunit of the classical protein phosphatase 1. However, two PPZ specific elements (the L1 loop and a C-terminal extra helix) were found in addition to several fine differences that explain the distinct regulation of the novel versus classical phosphatases and may provide a handle for the selection of PPZ directed specific phosphatase inhibitors [22].

Unfortunately, much less is known about the structure of the variable N-terminal domain, even though it may have significant contribution to the functioning of CaPpz1.Two lines of indirect evidence suggest that the N-terminal segment regulates salt tolerance: (i) The N-terminal domain contains a potential myristoylation site, and the mutation of this site in *Saccharomyces cerevisiae* ScPpz1 prevented the complementation of the characteristic LiCl tolerance of *ppz1* mutant, while the elimination of the whole N-terminal domain rendered the phosphatase ineffective in complementation tests [23]. (ii) In the *Debaryomyces hansenii* ortholog, DhPpz1, a Ser/Arg rich, short N-terminal sequence was identified that mediated the extreme salt tolerance of the *dhppz1* deletion mutant [24].

It has been recently reported that the phosphatase inhibitory effect of the so called “inhibitor-2” protein was masked by the N-terminal tail in the *C*. *albicans* CaPpz1 enzyme [22], and it was suggested that the flexible disordered N-terminal tail of the protein may fold back on the catalytic domain and may block the access of the protein substrates to the active site. In order to dissect the functional role of the N-terminal domain and of its different structural elements we designed an *in vitro* mutagenesis based strategy. By deleting large segments or smaller predicted functional units from CaPpz1 as well as by introducing precise point mutations we mapped the most important region of regulation to the first 16 amino acids of the phosphatase.

## Materials and methods

### Materials

Common chemicals and reagents were obtained from Sigma-Aldrich. The components of yeast culturing media as well as LiCl and caffeine were from VWR. Certified molecular biology agarose (BioRad), 1 Kb Plus DNA Ladder (Invitrogen), and GelRed (Biotium) was used in agarose gel electrophoresis. Gene specific oligonucleotide primers were ordered either from Sigma-Aldrich or from Integrated DNA Technology Incorporation. Vector specific primers were provided by UD-GenoMed Medical Genomic Technologies Ltd. Restriction endonucleases and alkaline phosphatase of Promega, together with T4 DNA ligase of Thermo Scientific were used in cloning experiments. Other, more specific materials are described in the next paragraphs.

### *In vitro* mutagenesis and DNA cloning

**Fig 1:**
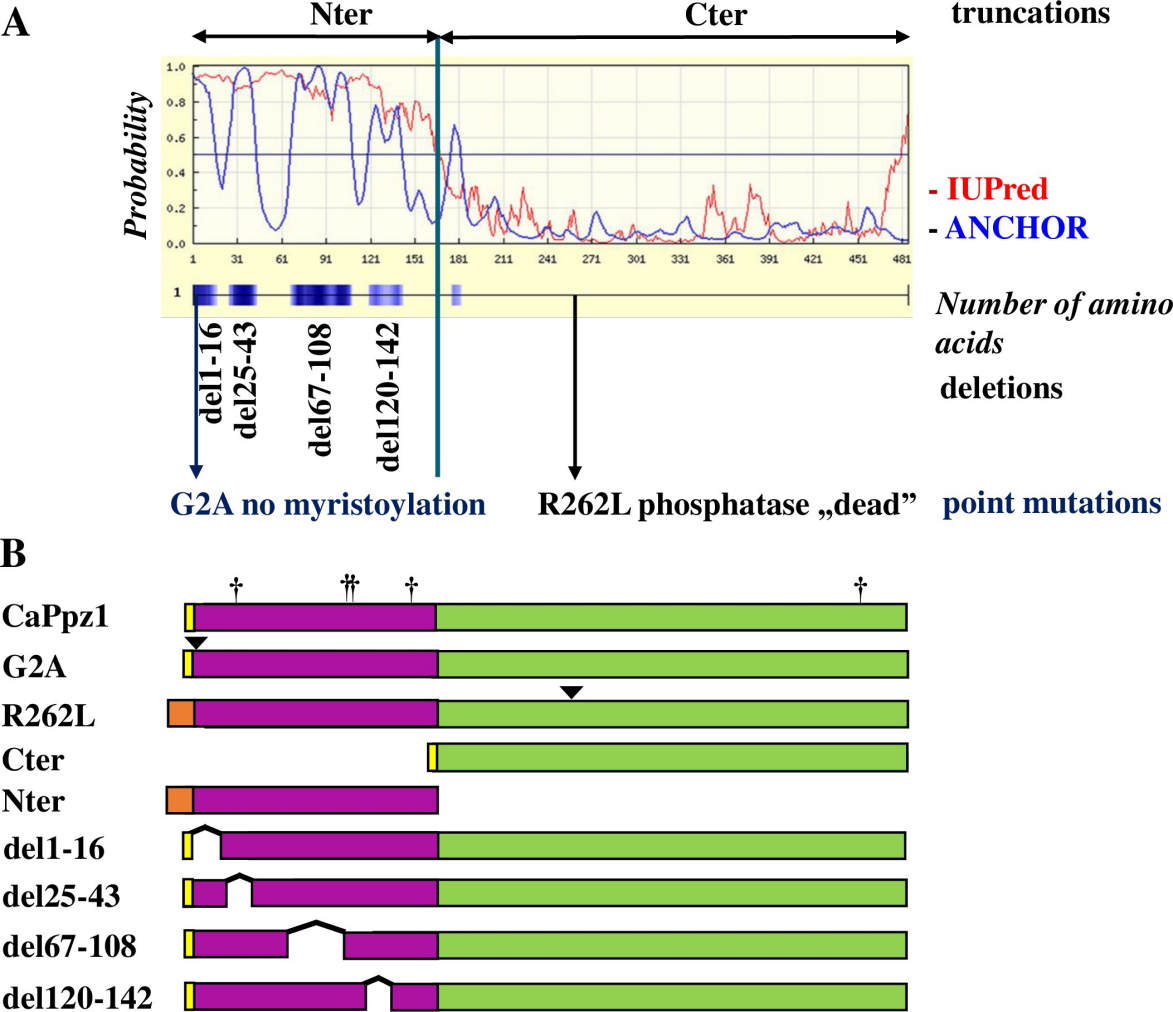
The rationale behind the *in vitro* mutagenesis of the CaPpz1 phosphatase. **A.** Bioinformatic analysis of the CaPpz1 protein. The IUPred (red line) and ANCHOR (blue line) software revealed the disordered regions (above 0.5 probability) and four main protein binding sites (blue boxes) in the N-terminal domain of CaPpz1. In the four CaPpz1 deletion mutants, these potential binding sites were eliminated. In addition, two point mutants (the not myristoylated G2A and the inactive R262L) were generated, and the two main domains of the protein (Nter and Cter) were expressed separately. **B.** Schematic representation of the bacterially expressed recombinant proteins. The yellow boxes show the residual part of the GST-tag that remains in the bacterially expressed proteins after Prescission protease cleavage, while the light brown boxes represent the 6xHis tag of the recombinant proteins (note that these features are not present in the proteins expressed in *S*. *cerevisiae*). The N-terminal domain is violet and the C-terminal domain is green. Point mutations are labeled with a triangle, and five unintentional S to L exchanges due to the special codon usage of *C*. *albicans* are indicated by crosses.

Mutations (see Fig 1) were generated in the wild type *CaPPZ1-3* allele [18], GenBank: GQ357913.1) with the QuikChange II XL Site-directed Mutagenesis Kit (Agilent Technologies) by using the pET28a(+)-CaPPZ1 plasmid target [19]. Mutagenic primers were designed by the QuikChange Primer Design Program (*www*.*agilent*.*com/genomics/qcpd**)* and are given in S1 Table. Nucleotides 73–129, 199–324, and 358–426 were eliminated from the target sequence (resulting in the mutations del25-43, del67-108, and del120-142, respectively) with the aid of the sense and antisense primer pairs listed in S1 Table. Deletion of amino acids 1–16 (del1-16) was made by the amplification of a truncated *CaPPZ1* sequence from pET28a(+)-CaPPZ1 by Phusion DNA polymerase (Thermo Fisher Scientific) with the CaPPZ1del1-16EcoRI and RevCaPPZCterXhoI primers. The N-terminal domain of CaPpz1 (Nter) coding sequence was produced by PCR amplification with the CaPPZNdeI and RevCaPPZNterXhoI primer pair and this amplicon was cloned in pET28a(+) using its NdeI and XhoI sites. The point mutation G2A was prepared in the YCplac111 and YEplac181 expression vectors carrying wild type *CaPPZ1-3* downstream of the *S*. *cerevisiae ScPPZ1* promoter [19] with the QuikChange II XL kit using the primers CaPPZG631Cfw and CaPPZG631rev.

For bacterial expression we decided to produce most of the recombinant proteins fused to an N-terminal GST-tag. That is why the ORFs of *CaPPZ1* and its mutants were transferred to pGEX-6P-1 (Amersham Biosciences-GE Healthcare) from their parent plasmids by a PCR based strategy. Full length *CaPPZ1*, as well as the mutant constructs for del25-43, del67-108, del120-142, and R262L were amplified with the primers CaPPZEcoRI and RevCaPPZXhoI; whereas the GlyAla.EcoRI and RevCaPPZCterXhoI primers were used for the G2A mutant (S3 Table). The mutagenized del1-16 PCR product was used directly for subcloning. All of the PCR products were ligated into pGEX-6P-1 *via* their primer encoded restriction sites (see S1 and S2 Tables).

For the expression of full length CaPpz1 and its mutants in *S*. *cerevisiae*, the appropriate coding regions were inserted in modified YCplac111 and YEplac181 plasmids that contained the *ScPPZ1* promoter [19]. The coding regions were inserted downstream of the *ScPPZ1* promoter between the XbaI and HindIII sites. To generate XbaI and HindIII sites for cloning we used a set of oligonucleotide primers listed in S2 Table. ORFs for *CaPPZ1*, del25-43, del67-108, del120-142, and R262L were amplified with the C1XbaI forward primer and C2HindIII reverse primer. For amplification of del1-16 and Cter the C1XbaIdel1-16 and CterXbaI forward primers were used (instead of C1XbaI) in combination with C2HindIII, while Nter was amplified with the C1XbaI and NterHindIII primer pair. Note that the G2A mutation was generated directly in the two *S*. *cerevisiae* plasmids.

The expression vector constructs were transformed into *Escherichia coli DH5α* or *XL10-Gold* cells. Plasmids were isolated with the aid of EZ-10 Spin Column Plasmid DNA kit (Bio Basic Canada Inc.) or Qiaquick gel extraction kit (Qiagen), and the fidelity of the inserted sequences was confirmed by DNA sequencing [25] with the primers listed in S3 Table.

The production of constructs for the C-terminal fragment of CaPpz1 (Cter) in pGEX-6P-1 [25] and for the R262L mutant in pET28a(+) [19] were described previously.

### Bacterial expression and purification of wild type and mutant CaPpz1 proteins

The isolated plasmids were transformed into *E*. *coli* BL21 (DE3)-RIL cells (Stratagene) that are more suitable for the bacterial production of yeast proteins. GST-tagged proteins were expressed and purified by Glutathione Sepharose 4B (GE Healthcare) affinity chromatography basically as reported earlier [26] with some modifications. The GST-tag was removed by Prescission Protease (GE Healthcare) treatment. For the detailed protocol and a representative preparation see the Supplementary Methods and S1A Fig. His-tagged Nter domain was expressed and purified by Ni-NTA agarose (Qiagen) chromatography as before [19] with some modifications. The complete procedure is given in the Supplementary Methods and a representative preparation is presented in S1B Fig.

### Characterization of the purified recombinant proteins

The bacterially expressed proteins were investigated by sodium dodecyl sulfate polyacrylamide gel electrophoresis (SDS-PAGE) according to [27] with PageRuler Prestained Protein Ladder (Fermentas) as molecular mass (Mr) standard. The Commassie Blue R250 stained gel images were recorded in the ChemiDoc Touch Imaging System (BioRad) and were analyzed by the Image Lab 5.2.1 (BioRad) software (S1 Fig). All of the recombinant proteins had the estimated apparent molecular masses close to the calculated theoretical values. Some of the CaPpz1 related bands were accompanied with bacterial proteins that we were not able to remove by extensive washing. Based on the densitometric scanning of the lanes the purity of the phosphatase preparations isolated on Glutathione Sepharose 4B matrix (S1A and S1C Fig) was in the range of 10–46%. On the other hand, the two recombinant proteins purified by Ni-NTA agarose chromatography were 70–90% and 10–20% pure (S1B and S1C Fig).

The protein phosphatase activity of the preparations was assayed as described previously [25]. The radioactive, ^32^P-labeled myosin light chain substrate was prepared by using [γ-^32^P]ATP (Institute of Isotopes Co., Ltd.), Mg^2+^ and myosin light chain kinase [28]. In control experiments we used mock purified protein extract prepared from *E*. *coli* BL21 (DE3)-RIL cells that were transformed with the empty pET28a(+) or pGEX-6P-1 vectors. The control samples liberated a negligible amount of radioactive phosphate from the substrate that was considered as background and was subtracted from the radioactivity measured with the recombinant protein preparations. Phosphatase activity was expressed in international units: 1 U of enzyme liberates 1 μmol phosphate in 1 minute. Protein concentration was measured [29] with bovine serum albumin (Sigma-Aldrich) as standard. The specific activity was calculated in mU/mg protein units by taking into consideration the purity of each recombinant protein preparation. Assays were conducted in 3 technical replicates with at least 3 independent preparations. The significance of the results was analyzed with the two-sided Student’s t-test of the Excel software (Microsoft Corporation).

### Expression and characterization of wild type and mutant CaPpz1 in *S*. *cerevisiae*

YCplac111 and YEplac181 plasmid constructs were transformed into the *ppz1* (*MATa his3Δ1 leu2Δ met15Δ ura3Δ ppz1*::*kanMX4)* phosphatase mutant *S*. *cerevisiae* strain. In control experiments the isogenic BY4741 (*MATa his3Δ1 leu2Δ met15Δ ura3Δ*) strain was transformed with the empty plasmids. The transformation, as well as the culturing and testing of the transformants in drop tests were performed as earlier [19]. The LiCl sensitivity of the transformants was also determined in liquid YPD medium by the adaptation of the protocol described in [19] with the modification that the yeast cells were cultivated at 28°C under continuous shaking at 180 rpm. Optimal growth conditions and LiCl concentration dependence were determined in pilot experiments (S2 Fig). We found that the transformation did not affect the growth rate of the wild type and *ppz1* cells and we selected 19 h culturing in the presence of 50 mM LiCl for the subsequent investigations.

## Results

### Experimental design of the *in vitro* mutagenesis

To identify potentially important segments in CaPpz1 its translated protein sequence was analyzed by structure prediction tools (Fig 1A). According to the IUPred algorithm [30] the intrinsically disordered N-terminal domain (Nter) of the phosphatase comprises amino acids 1–171, while the globular C-terminal catalytic domain (Cter) contains residues 172–485 [22].

The first amino acid in the translated coding sequence is the initiating Met, which is eliminated from the mature protein, and the second amino acid is Gly, a potential N-terminal myristoylation site (https://web.expasy.org/myristoylator/). Note that the essential Arg that is required for enzyme activity is located at position 262. The ANCHOR software [31] predicted four potential protein binding segments between the amino acid residues 1–16, 25–43, 67–108, and 120–142 in the N-terminal domain. The role of these regions has not been addressed yet. In a recent publication the binding site of the Hal3 regulatory subunit of *S*. *cerevisiae* Ppz1 was mapped to a specific region of the catalytic domain [32]. Since Hal3 and its paralogs tend to bind to the C-terminal region of the phosphatase it is possible that the potential binding segments of the N-terminal domain interact with some, up till now unidentified protein partners, and/or are responsible for the binding of the N-terminus to the catalytic domain of the same protein.

To dissect the role of the predicted structural elements inside the N-terminal domain of CaPpz1 we designed a mutagenesis based strategy (Fig 1A). The separated Nter and the Cter domains (truncations), the deletion mutants: del1-16, del25-43, del67-108, and del120-142 (devoid of one of the potential protein binding sites) as well as two point mutants were investigated in this study. We generated a G2A mutant to destroy the N-terminal myristoylation site [23] to test if the consequences of the del1-16 deletion were associated with the lack of myristoylation, or were caused by the elimination of additional functional elements, since a search with ELM (The Eukaryotic Linear Motif resource for Functional Sites in Proteins, http://elm.eu.org/search.html) predicted a WDR45 binding motif and several phosphorylation sites for CDK1 and GSK protein kinases within the deleted region. As a negative control we utilized the R262L mutant that was rendered catalytically “dead” by the exchange of the essential Arg residue in the catalytic domain [19, 23]. It should be mentioned that due to the different codon usage of *C*. *albicans* versus *E*. *coli* and *S*. *cerevisiae* a maximum of five unintentional Ser to Leu mutations were introduced into the expressed proteins (Fig 1B), but these mutations were considered “silent” based on previous studies [19].

### *In vitro* effects of the mutations

**Fig 2:**
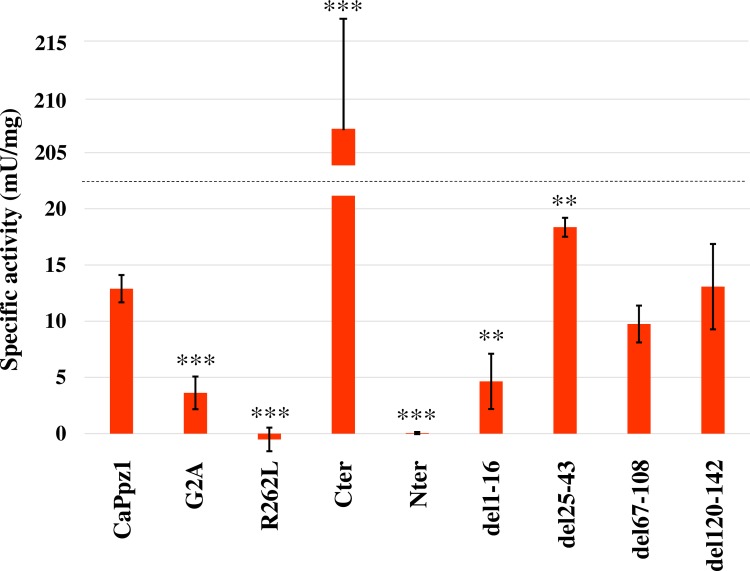
The role of the CaPpz1 N-terminal domain in phosphatase activity. The *in vitro* phosphatase activity of the bacterially expressed purified recombinant wild type and mutant CaPpz1 proteins was determined with ^32^P-labelled myosin light chain substrate. The mean and standard deviation of 3–4 independent assays is shown, ** indicates p<0.01 and *** labels p<0.001 significance.

For testing the effects of mutations on enzyme activity, originally we planned to express 6xHis-tagged recombinant proteins in *E*. *coli* according to [19]. However we noticed that some of the mutant proteins were produced with low efficiency, and were difficult to purify with this tag. To circumvent this problem—with the exception of Nter and R262L - we expressed most of the mutant proteins as well as the full length CaPpz1 as GST-tagged fusion proteins (Fig 1B). To this end pGEX-6P-1 based vector constructs were generated, the GST-fusion proteins were purified by Glutathione Sepharose 4B affinity chromatography, and the GST-tag was removed with Prescission protease. The Nter domain and the R262L mutant were expressed with an N-terminal 6xHis-tag, and were purified by Ni-agarose affinity chromatography. The purity of the preparations was determined by SDS-PAGE (S1 Fig). The phosphatase activity of these recombinant proteins was assayed *in vitro* (Fig 2).

As expected the N-terminal domain alone and the R262L mutant were inactive. The del67-108 and del120-142 mutations did not cause any significant change in the specific activity in comparison to the wild type phosphatase. Deletion of amino acids 1–16 as well as the G2A point mutation within this region significantly reduced the activity of CaPpz1. In contrast, the del25-43 mutant had a moderately elevated activity. It can be hypothesized that the 25–43 peptide region may directly interact with the C-terminal catalytic domain, and its elimination may lift the inhibitory effect of the N-terminal tail on the catalytic activity. However, without knowing the 3D structure of full length CaPpz1, it is hard to confirm the mechanism and predict the significance of this moderate change in the *in vitro* enzyme activity. A large, nearly 16-fold increase of specific activity was measured when the free catalytic domain of CaPpz1 (Cter) was assayed with the radioactive protein substrate.

### *In vivo* effects of the mutations

For revealing the physiological roles of mutations described in Fig 1. we used a testing system described before [19]. Previously we reported that CaPpz1 partially rescued the caffeine sensitivity and salt tolerance phenotypes of a *S*. *cerevisiae* mutant lacking the orthologous *ScPPZ1* gene. Therefore, we analyzed the *in vivo* effects of CaPpz1 mutations by assessing their ability to complement the *ppz1* deletion mutant. In our experiments the full length CaPpz1 and its mutants were expressed in *ppz1* cells under the control of the *ScPPZ1* promoter either from low copy number centromeric YCplac111-based plasmids or from high copy number episomal YEplac181-based constructs and the caffeine sensitivity as well as the salt tolerance of the transformants were tested.

#### Caffeine sensitivity tests

**Fig 3:**
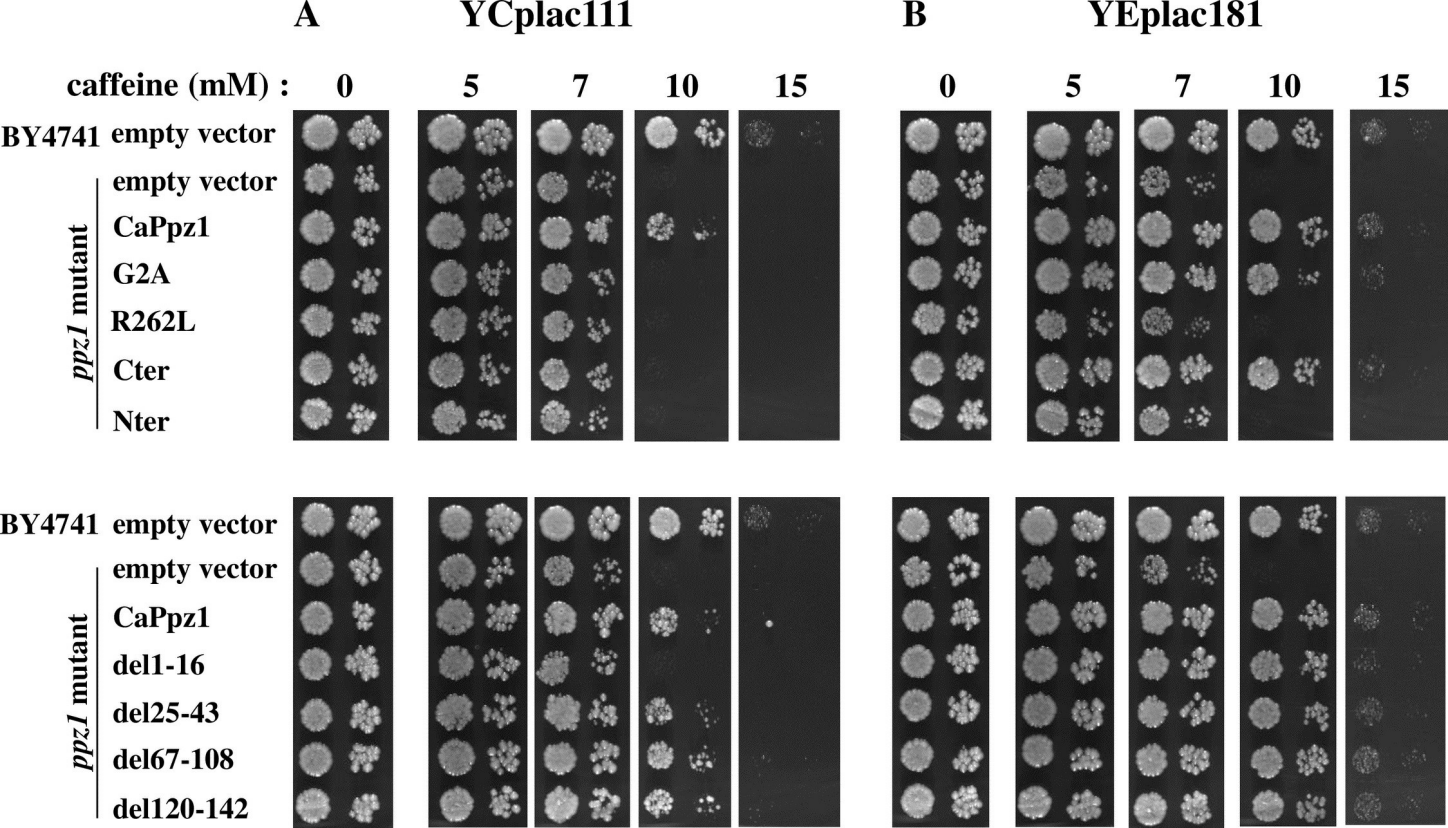
Complementation of the caffeine sensitivity of *ppz1 S*. *cerevisiae* cells by the expression of mutant CaPpz1 proteins. Wild type BY4741 *S*. *cerevisiae* strain or its isogenic *ppz1* deletion mutant strain were transformed with empty plasmids as a negative control. The mutant strain was also transformed with YCplac111 **(A)** or YEplac181 **(B)** carrying wild type or mutant *CaPPZ1* inserts (that are labeled by the names of the encoded proteins). Yeast cells were plated on YPD in the absence or in the presence of increasing concentrations of caffeine and the growth rate of the yeast cells was determined in spot tests. 3 x 10^3^ and 3 x 10^2^ cells were spotted on YPD plates, which were photographed after 72 h incubation. Representative results of 3 independent experiments are shown.

The caffeine sensitivity of the *S*. *cerevisiae ppz1* deletion mutant can be attributed to the involvement of ScPpz1 in the cell wall biosynthesis and in the cell integrity pathway [17]. Complementation of this phenotype by the expression of *C*. *albicans* CaPpz1 in the *ppz1* mutant yeast cells has been reported [19]. Based on these observations we studied the efficiency of the mutated CaPpz1 forms in complementation experiments (Fig 3).

Expression of CaPpz1 from YCplac111 (Fig 3A) markedly reduced the caffeine sensitivity of the *ppz1* strain, and the complementations by the del25-43, del67-108, and del120-142 CaPpz1 deletion mutants were similar to those of wild type CaPpz1. On the other hand, del1-16, G2A, Nter and R262L mutants as well as the Cter domain were almost ineffective. To test the possibility that the small effects of some expressed proteins were associated with the low efficiency of expression from centromeric plasmids, we repeated the experiments with high copy number YEplac181 episomal plasmid-based constructs (Fig 3B). In the control experiment the more efficient expression of CaPpz1 eliminated caffeine sensitivity nearly completely and in the complementation tests only the inactive Nter and the phosphatase “dead” R262L mutant remained ineffective.

#### Salt tolerance tests

**Fig 4:**
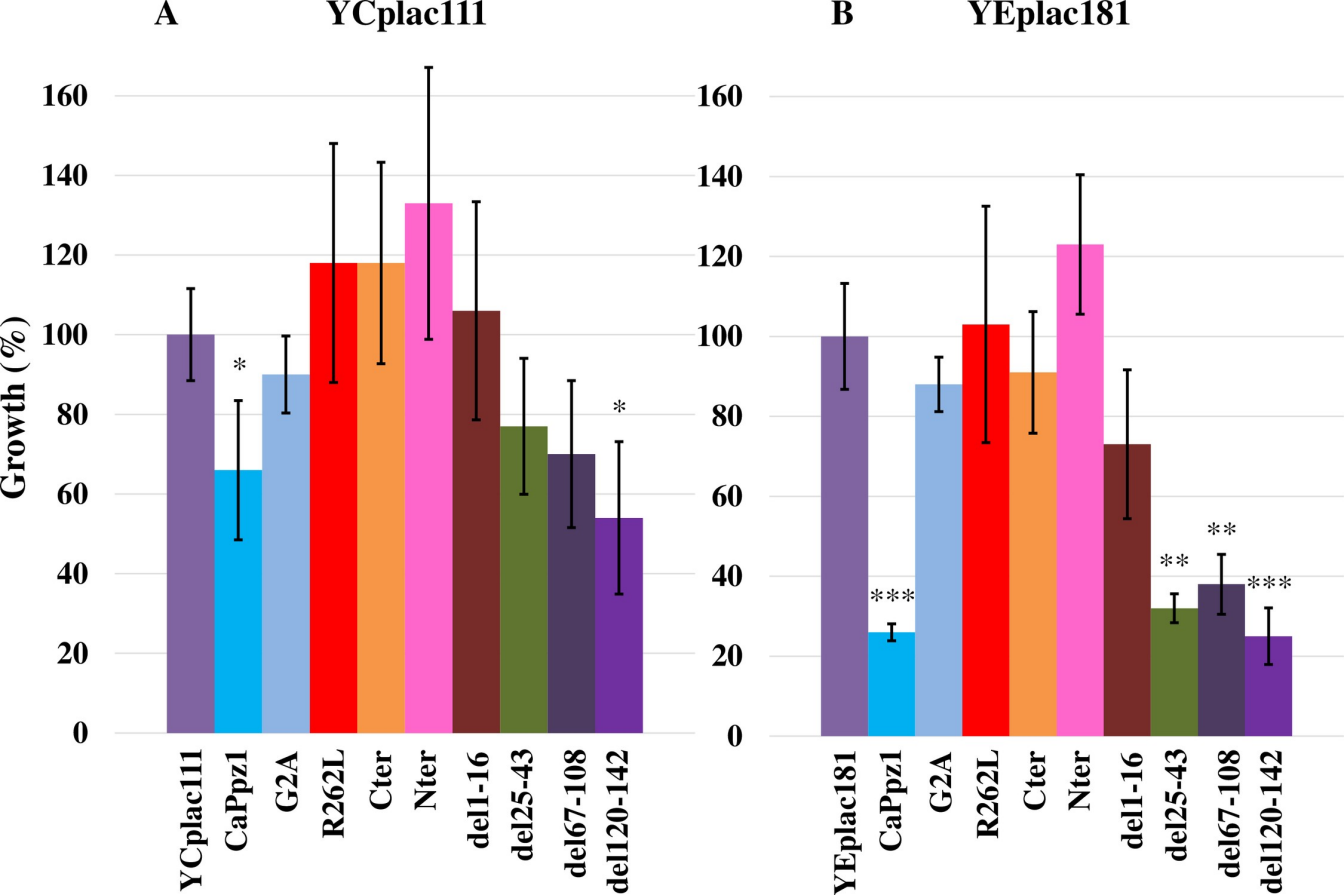
Complementation of the salt tolerance of *ppz1 S*. *cerevisiae* cells by the expression of mutant CaPpz1 proteins. The phosphatase mutant *ppz1 S*. *cerevisiae* strain was transformed either with empty plasmids or with YCplac111 **(A)** or YEplac181 **(B)** carrying wild type or mutant *CaPPZ1* inserts as in Fig 3. After establishing optimal assay conditions the effect of 50 mM LiCl on the transformants was determined after 19 h of cultivation. The growth of the cells containing empty plasmids was taken as 100%. Mean and standard deviation of 3 independent experiments are shown.

In *S*. *cerevisiae* ScPpz1 regulates salt homeostasis [17]. Its deletion results in tolerance against the monovalent cations Na^+^ and Li^+^ due to the elevated expression of the ENA1 sodium transporter [33]. The LiCl tolerant mutant can be complemented by the expression of CaPpz1 [19] that allowed us to test the effect of the mutant *C*. *albicans* proteins basically as described in the previous section. However, in the spot tests we detected only barely visible changes upon the transformation of the *S*. *cerevisiae ppz1* mutant cells with YCplac111 plasmids, and did not get photogenic data with the YEplac181 vector either (not documented data). That is why we changed the qualitative detection method and switched to the liquid cultures that can be characterized quantitatively by measuring their turbidity at 620 nm (Fig 4).

The transformation with low copy number YCplac111 constructs resulted in incomplete complementation of the characteristic Li-ion tolerance, only the wild type CaPpz1 and the del120-142 mutant exhibited a significant reduction of growth rate in the presence of LiCl (Fig 4A). Overexpression from YEplac181 provided more clear-cut results (Fig 4B). Expression of CaPpz1 at higher level restored LiCl sensitivity effectively, and the deletion mutants: del25-43, del67-108, and del120-142 had a similar significant effect. It has to be stressed that we were not able to see any difference between the wild type and the del25-43 mutant in these assays. In contrast, the overexpression of the point mutants (G2A and R262L), the truncated mutants (Nter and Cter) and the del1-16 mutation resulted in no significant change.

## Discussion

Previously two similar *in vitro* mutagenesis based structure-function studies of fungal protein phosphatase Z enzymes have been published [23, 24]. In their pioneering work Clotet et al. [23] investigated the ScPpz1 isoenzyme of *S*. *cerevisiae* by deleting two large segments of the N-terminal domain which were conserved between the ScPpz1 and ScPpz2 paralogs. Minhas et al. [24] deleted six short Ser rich low complexity regions from the N-terminal domain of the *D*. *hansenii* DhPpz1 phosphatase. In the present study we investigated the role of four potential protein binding sites in the disordered N-terminal half of the *C*. *albicans* CaPpz1 (Fig 1). Due to the different selection strategies of deletions and because of the low level of sequence similarity between the N-terminal domains of the experimental organisms (S3 Fig) it seems to be difficult to correlate our present results with the published data. However, despite of the differences in the length and diversity of amino acid sequences, the intrinsically unstructured nature of the N-terminal domains have been conserved (S4 Fig) and some of the deleted regions, as well as the point mutations and truncations correspond to each other in the three proteins. Thus our experimental design offers a possibility to test the functional conservation of some regions in the N-terminal half of the phosphatases.

Our *in vitro* phosphatase assay demonstrated that the deletion of amino acids 1–16 as well as elimination of the myristoylation site (G2A) resulted in a decreased CaPpz1 activity (Fig 2). In correlation with this finding the bacterially expressed *S*. *cerevisiae* Ppz1 G2A mutant also had somewhat lower phosphatase activity (measured with myelin basic protein substrate) than the recombinant wild type enzyme [23]. Consequently the short peptide, containing Gly2 at the very N-terminal end is required for the folding and/or activation of CaPpz1. Note that the recombinant Gly2 cannot be myristoylated, since it is still attached to the residual 7 amino acid residues of the N-terminal GST-tag that is left behind after the scission by Precission protease. It is possible that the Gly residue acts as a hinge in the wild type protein, and its mutation in G2A or deletion in del1-16 places the residual peptide in an unfavorable position.

On the other hand, the elimination of the whole N-terminal domain resulted in a radical elevation of the CaPpz1 phosphatase activity (Fig 2). This fact has already been mentioned in the literature, but was not documented [22], and deserves a detailed discussion, since it contradicts the result telling that the C-terminal domain of *S*. *cerevisiae* ScPpz1 is less active than the full length enzyme [23]. There are several differences between our assay conditions, but we believe that the most important factor is that Clotet et al. [23] measured the activity of a GST-fusion protein while we removed the bulk of the GST-tag from Cter. Previously it was reported that the GST-tag had no effect on the kinetic properties of the recombinant *S*. *cerevisiae* Ppz1 [34], however the same test has not been performed with the C-terminal domain. The main difference between the two recombinant proteins is that when the 26 kDa GST-tag is attached to N-terminus of the catalytic domain it may prevent the binding of a protein substrate to the catalytic site, while in the tagged full length Ppz1 protein the N-terminal domain blocks the access of the catalytic cleft anyway and the GST-tag has no additional effect on the activity.

The hypothesis of spatial hindrance of CaPpz1 activity by the Nter was suggested earlier [22] and is supported by several lines of evidence. The elevated specific activity of Cter against a protein substrate (Fig 2) and the higher sensitivity of Cter against the inhibitor 2 protein [22], in comparison to full length CaPpz1 have already been mentioned. Furthermore, both of the potential CaPpz1 inhibitor proteins of *C*. *albicans* (CaCab3 and CaHal3) bound more effectively to the C-terminal domain than to the full length version of the phosphatase according to several *in vitro* protein-protein interaction assays [26]. Finally, Posas et al. [34] reported that the specific activity increased more than 6-fold when the recombinant *S*. *cerevisiae* ScPpz1 was treated with trypsin, indicating that the proteolytic digestion eliminated some N- or C-terminal peptides which repressed catalytic activity. Thus it is likely that the N-terminal region plays an inhibitory role in PPZ phosphatases. It should be noted that the externally added N-terminal domain did not affect the activity of either the full length CaPpz1 or its separate C-terminal catalytic domain (K. Petrényi unpublished results). Thus the covalent linkage connecting the two domains is imperative for the suggested regulation of CaPpz1 activity by its N-terminal tail.

From our complementation experiments (Figs 3 and 4) we conclude that the deletions del25-43, del67-108, and del120-142 have no effect on the investigated phenotypes of the *ppz1* mutant. In agreement with these results the large Δ17–193 deletion of ScPpz1 [23] (that covers all of the above CaPpz1 deletions) and the small Δ162–133 mutation of DhPpz1 [24] (which is related to del120-142) had no effect on the caffeine sensitivity and salt tolerance of the corresponding phosphatase mutants.

On the other hand, the inactive Nter and R262L mutants failed to complement the *ppz1* mutation (Figs 3 and 4). In accord with the latter finding an analogous Arg to Leu mutation in ScPpz1 also resulted in loss of phosphatase activity and loss of function in caffeine sensitivity and LiCl tolerance [23]. Obviously, the phosphatase activity is an indispensable requirement for the cell wall integrity and osmotic stability related functions of PPZ phosphatases. Apparently this conclusion is in variance with the finding that low copy expression of the highly active Cter had no effect in both of our physiological experiments (Figs 3A and 4A). This is in correlation with an earlier report [23] stating that the C-terminal domain of ScPpz1 was inactive in similar tests. It was also reported in the same article [23] that C-terminal domain of ScPpz1 was dislocated in budding yeast suggesting that the N-terminal domain was essential for the localization of the phosphatase into a particulate fraction that can be sedimented by high speed centrifugation. Thus it is likely that the active Cter of CaPpz1 was inefficient as it was not localized properly in *S*. *cerevisiae*. Interestingly the overexpression of Cter overcame the consequences of the defective localization in the caffeine sensitivity (Fig 3B) but did not help significantly in the salt tolerance test.

The distinct structural requirements for the two tested phenotypes were also noted in two additional cases. The del1-16 and G2A mutants behaved similarly in the caffeine sensitivity tests, at lower expression level they were not effective, but complemented the mutant phenotype when they were overexpressed (Fig 3). An analogous G2A mutation of ScPpz1 was complementing efficiently at low copy expression [23] suggesting that the heterologous CaPpz1 mutants function less effectively in *S*. *cerevisiae* than the similar authentic phosphatase mutant. In contrast the two mutants did not restore salt sensitivity neither at lower nor at higher expression levels (Fig 4). The nearly identical effects of the del1-16 deletion and G2A point mutation indicate that the defective myristoylation of the N-terminal Gly was the dominant common factor that determined the functions of the two mutants. It has been demonstrated earlier that the N-terminal Gly of ScPpz1 was effectively myristoylated *in vitro*, and this post-synthetic modification did not localize the enzyme to membranes *in vivo* [23]. The replacement of Gly2 by Ala did not change the particulate localization of the ScPpz1 either [23], but it is still possible that the N-terminal myristoylation of the PPZ enzymes is important in protein-protein interactions. The differential effect of mutations suggests that the N-terminal myristoylation is more important for the salt tolerance than for the caffeine sensitivity phenotype of the *ppz1 S*. *cerevisiae* mutant strain.

## Conclusions

From our structure-function study we conclude that both the enzyme activity of the C-terminal domain and the proper localization of the enzyme by its N-terminal domain are essential for the physiological functions of the CaPpz1 phosphatase. The first 16 amino acid residues, including the Gly2 myristoylation site, of the CaPpz1 phosphatase are especially important as the deletion of this region or the G2A mutation significantly decreased the specific activity of the enzyme *in vitro* and also diminished the ability of the mutant proteins to complement the caffeine sensitivity and salt tolerance of the *ppz1 S*. *cerevisiae* deletion mutant *in vivo*. Our results are in good agreement with the data of Clotet et al. [23] indicating that the structure and the functional role of the very N-terminal residues in the *S*. *cerevisiae* and *C*. *albicans* PPZ phosphatases have been well conserved during evolution despite the fact that the overall amino acid sequences of the N-terminal domains are quite divergent in the two species (S3 and S4 Figs). The correlation between our work and the paper of [24] is less obvious. Although we agree that the Ser/Arg rich sequence (residues 23–32 in *C*. *albicans*) may be important since the del25-43 mutant (in which a nearly completely overlapping peptide region was deleted) exhibited higher phosphatase activity, however, we were not able to demonstrate the effect of this deletion in the LiCl sensitivity tests. Due to these circumstances we confirmed the functional significance of the 1–16 peptide region but did not find convincing evidence for the functioning of additional predicted protein binding regions in the N-terminal domain of CaPpz1.

## Supporting information

S1 Text Supplementary Methods. (DOCX)

S1 Table Oligonucleotide primers used for mutagenesis. (DOC)

S2 Table Oligonucleotide primers used for cloning. (DOC)

S3 Table Gene specific primers used for DNA sequencing. (DOC)

S1 Fig Purification and testing of recombinant proteins. **A.** Purification of recombinant wild type CaPpz1. Bacterial cell extract was separated to supernatant and pellet by centrifugation and the GST-tagged CaPpz1 was purified from the supernatant on Glutathione Sepharose in batch mode. The bulk of bacterial proteins which did not bind to the resin (unbound proteins) were removed by centrifugation. Weakly bonded proteins were eliminated by washing in six steps. The first, second, fifth, and sixth wash fractions were tested in the gel. The resin with bonded GST-tagged protein was treated with Prescission protease. Fractions containing the cleaved off CaPpz1protein were eluted with five portions of the protease buffer. The third fraction (boxed) was used for further assays. The efficiency of the cleavage was demonstrated by the analysis of an aliquot of Glutathione Sepharose resin before and after protease treatment and elution. The SDS-PAGE analysis of a representative preparation is shown. St. indicates PageRuler Pre-stained Protein Ladder that was used for the estimation of the apparent molecular mass (Mr) of selected bands. The apparent Mr of CaPpz1 is 58.9 kDa that is somewhat more than the theoretical 55.5 kDa value. **B.** Purification of the N-terminal domain (Nter) of CaPpz1. His-tagged Nter was purified on Ni-NTA-agarose. The supernatant and pellet resulting from centrifugation of the cell extract, unbound proteins, as well as the wash and eluted fractions were analyzed by SDS-PAGE as in S1 Fig. The apparent Mr of Nter is 20 kDa in agreement with the theoretical value of 20.9 kDa. The boxed fraction was used for further assays. **C.** Testing the purity of mutant CaPpz1 proteins. Recombinant proteins G2A, Cter, del1-16, del25-43, del67-108, and del120-142 were purified on Glutathione Sepharose as the wild type CaPpz1 phosphatase (panel A). Three eluted fractions and the residual resin after cleavage and elution were subjected to SDS-PAGE analysis. The boxed fractions were used for phosphatase assays. The R262L mutant protein was purified on Ni-NTA-agarose as the Nter domain (panel B) and three eluted fractions as well as the residual resin were analyzed by SDS-PAGE. Apparent (red) and theoretical (black) molecular masses (Mr) of the recombinant proteins are given below the panels. The theoretical Mr was calculated with the ExPASy compute pI/Mw tool https://web.expasy.org/compute_pi/. Boxes indicate the band of the recombinant proteins in the fractions that were used for the phosphatase assays. A representative of at least three independent preparations is shown in the figure.
(TIF)

S2 Fig Determination of optimal conditions for testing salt tolerance. **A.** Wild type BY4741 and *ppz1* deletion mutant *S*. *cerevisiae* cells were cultivated without any addition (blue bars), and after transformation with empty or CaPpz1 coding YCplac111 (green) and YCplac181 (red) plasmids. The turbidity (OD_620_) of triplicate cultures was determined after 19 h incubation. The means of two independent experiments is shown. **B.** The *ppz1* mutant (triangles) and BY4741 control (circles) yeast strains were transformed either with empty (empty symbols) or with CaPpz1 harboring (full symbols) plasmids and were cultivated in the presence of increasing concentrations of LiCl. After 19 h of incubation the optical density of triplicate cultures was measures at 620 nm. The relative growth of the samples cultivated in the absence of LiCl was taken as 100%. The means of two independent experiments is depicted.
(TIF)

S3 Fig Comparison of *S*. *cerevisiae* ScPpz1, D. *hansenii* DhPpz1 and *C*. *albicans* CaPpz1 protein sequences. The amino acid sequences that were investigated by Clotet et al., 1996, Minhas et al., 2012, and in the present work were aligned by the Clustal Omega multiple sequence alignment tool (https://www.ebi.ac.uk/Tools/msa/clustalo/). Green background indicates deletions and pink highlights point mutations. Yellow insert marks the deletion in ScPpz1 that was studied by Minhas et al, 2012. The light blue line above the sequences labels the C-terminal domain of CaPpz1, dark blue extensions mark the limits of the same domain in ScPpz1. Stars under the sequences indicate amino acid residue identities, spot show similarities.
(PDF)

S4 Fig Overview of in vitro mutagenesis studies of fungal protein phosphatase Z enzymes. Protein sequences shown in S3 Fig were analyzed by the IUPred2 and ANCHOR2 combined web interface (https://iupred2a.elte.hu/) for protein disorder (red lines) and protein binding regions (blue lines). The graphs were aligned along the conserved catalytic domains. Green bars and a yellow insert indicate deletions, pink stars show point mutations, and blue bars delimit the C-terminal domains as in panel A. Full black lines connect structurally related elements and dashed black lines connect the borders of related regions.
(TIF)
