# Supplementary material for: Dissection of the regulatory role for the N-terminal domain in Candida albicans protein phosphatase Z1
Source: PLoS One. 2019 Feb 1;14(2):e0211426. doi: 10.1371/journal.pone.0211426 (PMC6358084; doi:10.1371/journal.pone.0211426)
Supplement: S1 Text — (DOCX) [file pone.0211426.s001.docx]

**Supplementary Methods**

***Expression and Preparation of GST-tagged Recombinant Proteins***

Wild type and active mutant CaPpz1 phosphatases were expressed in LB broth supplemented with 0.5 mM MnCl_2_, in the presence of 100 μg/ml ampicillin and 34 μg/ml chloramphenicol. Expression of proteins with N-terminal GST-tag was induced with 0.5 mM IPTG for 3 hr at 18 °C. Recombinant GST-tagged proteins were purified on Glutathione Sepharose 4B (GE Healthcare) in batch mode. Cells were collected from a 1 liter culture and were sonicated with Branson Sonifier 250 in a lysis buffer containing 50 mM Tris-HCl (pH 7.5), 150 mM NaCl, 1 mM MnCl_2_, 2 mM dithiotreitol, 10% glycerol, 1% Triton X-100, 0.5 mM phenylmethylsulfonyl fluoride, 0.5 mM benzamidine, and EDTA-free cOmplete protease inhibitor cocktail (Roche). Cell lysate was centrifuged for 20 min at 5500 rpm and the resulting supernatant was incubated with 500 μl of 80% Glutathione Sepharose slurry for 3 hours under gentle agitation. The solution of unbound proteins was removed by centrifugation according to the manufacturer’s instructions. The resin was washed with 1 ml of lysis buffer three times, then with 1 ml of lysis buffer without Triton X-100 two times, and finally, with 1 ml of a lysis buffer without Triton X-100 but supplemented with 1 mM EDTA. The GST-tag was removed and the recombinant proteins were eluted according to the recommendations of GE Healthcare except that the cleavage step was done longer. First the GST-protein-bound resin was incubated for two hours with 4 units of Prescission Protease (GE Healthcare) in 250 μl of Prescission Protease buffer supplemented with 1 mM MnCl_2_ and 2 mM dithiothreitol. Then an aliquot of fresh protease was added, and the digestion was continued overnight. The eluate fraction was collected, and the resin was eluted further with 250 μl aliquots of the above buffer but without the protease. A representative preparation is shown S1 Fig A and the purities of the additional preparations are documented in S1 Fig C.

***Expression and Preparation of 6xHis-tagged Recombinant Proteins***

The His-tagged N-terminal domain of CaPpz1 (Nter) and the R262L inactive phosphatase mutant were produced in *E. coli* BL21 (DE3)-RIL cells similarly to the previous chapter with the exception that the expression of R262L was induced by the addition of 0.4 mM IPTG while the induction of Nter was carried out with 0.8 mM IPTG. After an overnight incubation at 18°C, cells were lysed in a buffer containing 25 mM Tris-HCl (pH 7.5), 250 mM NaCl, 1 mM MnCl_2_, 0.1 % Triton X-100, 5 mM imidazole, 1 mM 2-mercaptoethanol, 1 mM benzamidine, 1 mM phenylmethylsulfonyl fluoride, and EDTA-free cOmplete protease inhibitor cocktail (Roche). The supernatant of the bacterial extract was gently shaken with 800 μl of Ni-NTA agarose (Qiagen) equilibrated in the lysis buffer. The slurry was then transferred into a mini column. The flowthrough fraction containing the unbound proteins was collected for analysis, and the resin was washed two times with 10 ml of a washing buffer containing 25 mM Tris-HCl (pH 7.2), 800 mM NaCl, 1 mM MnCl_2_, 20 mM imidazole, 1 mM 2-mercaptoethanol, 1 mM benzamidine, 1 mM phenylmethylsulfonyl fluoride, and EDTA-free cOmplete protease inhibitor cocktail. Then the resin was washed two times with a second washing buffer that was similar to the first one except that it contained 200 mM NaCl and 30 mM imidazole. Tagged proteins were eluted with 5 ml of 200 mM imidazole dissolved in the second washing buffer. For a representative preparation of Nter see S1 Fig B, and for the characterization of the R262L mutant consult S1 Fig C.
