# Supplementary material for: Dissection of the regulatory role for the N-terminal domain in Candida albicans protein phosphatase Z1
Source: PLoS One. 2019 Feb 1;14(2):e0211426. doi: 10.1371/journal.pone.0211426 (PMC6358084; doi:10.1371/journal.pone.0211426)
Supplement: S1 Table — (DOC) [file pone.0211426.s002.doc]

**Table S1. Oligonucleotide primers used for mutagenesis**.

| **Mutation** | **Primer name** | **Primer sequence (5’→3’)*** | **Plasmid** |
| --- | --- | --- | --- |
| **del1-16** | CaPPZ1del1-16EcoRI | AACGAATTCACTAATAATTCCACAAGATC | pGEX-6P-1 |
|  | RevCaPPZCterXhoI | TTTACTCGAGCTTTATGTAGATTTCTTTC |  |
| **del25-43** | CaPPZdel73-129 | CTAATAATTCCACAAGATCTACTCAACAGCAATCAGACTCACAACA | pET28a(+) |
|  | CaPPZdel73-129-AS | TGTTGTGAGTCTGATTGCTGTTGAGTAGATCTTGTGGAATTATTAG |  |
| **del67-108** | CaPPZdel199-324 | GCAATCACAATCACAATCACAACATCTGCAGCAACAACAACAAC | pET28a(+) |
|  | CaPPZdel199-324-AS | GTTGTTGTTGTTGCTGCAGATGTTGTGATTGTGATTGTGATTGC |  |
| **del120-142** | CaPPZdel358-426 | GCAACAACAACAACAGCCAGGCTCAACATCTCACAAGT | pET28a(+) |
|  | CaPPZdel358-426AS | ACTTGTGAGATGTTGAGCCTGGCTGTTGTTGTTGTTGC |  |
| **Nter** | CaPPZNdeI | AAACATATGGGTTCTAATTCATCTAAATCAG | pET28a(+) |
|  | RevCaPPZNterXhoI | TTTTCTCGAGTCAAGTATTAGAATTTGTACTTAGTGATG |  |
| **G2A** | CaPPZG631Cfw | TCTTTCCTTCTAGATCAAAATGGCTTCTAATTCATCTAAATCAGCTC | Modified**  YCplac111  and YEplac181 |
|  | CaPPZG631Crev | GAGCTGATTTAGATGAATTAGAAGCCATTTTGATCTAGAAGGAAAGA |  |

*The underlined sequences correspond to the restriction sites indicated in the names.

**Modified YCplac111 and YEplac181 contain the *S. cerevisiae* *PPZ1* promoter.
