# Supplementary material for: Dissection of the regulatory role for the N-terminal domain in Candida albicans protein phosphatase Z1
Source: PLoS One. 2019 Feb 1;14(2):e0211426. doi: 10.1371/journal.pone.0211426 (PMC6358084; doi:10.1371/journal.pone.0211426)
Supplement: S2 Table — (DOC) [file pone.0211426.s003.doc]

**Table S2.** **Oligonucleotide primers used for cloning.**

| **Primer name** | **Primer sequence (5’→3’)*** | **Destination** |
| --- | --- | --- |
| CaPPZEcoRI | ATAGAATTCATGGGTTCTAATTCATC | pGEX-6P-1 |
| RevCaPPZXhoI | CCTCTCGAGCTTTATGTAGATTTC | pGEX-6P-1 |
| GlyAla.EcoRI | ATAGAATTCATGGCTTCTAATTCATC | pGEX-6P-1 |
| RevCaPPZCterXho1 | TTTACTCGAGCTTTATGTAGATTTCTTTC | pGEX-6P-1 |
| C1XbaI | GCTCTAGATCAAAATGGGTTCTAATTCATCTAAATC | Modified YCplac111 and YEplac181** |
| C2HindIII | GTAAGCTTTATGTAGATTTCTTTCTTTCTTGTTTTTC | Modified YCplac111 and YEplac181** |
| C1XbaIdel1-16 | GCTCTAGATCAAAATGACTAATAATTCCACAAGATCTACTAGG | Modified YCplac111 and YEplac181** |
| CterXbaI | GCTCTAGATCAAAATGATAGATTCATTAATTGATAAATTATTGAA | Modified YCplac111 and YEplac181** |
| NterHindIII | TTAAGCTTTCAAGTATTAGAATTTGTACTTAGTGATGAAG | Modified YCplac111 and YEplac181** |

*The underlined sequences correspond to the restriction sites indicated in the names.

**Modified YCplac111 and YEplac181 contain the *S. cerevisiae* *PPZ1* promoter.
