# Supplementary material for: Dissection of the regulatory role for the N-terminal domain in Candida albicans protein phosphatase Z1
Source: PLoS One. 2019 Feb 1;14(2):e0211426. doi: 10.1371/journal.pone.0211426 (PMC6358084; doi:10.1371/journal.pone.0211426)
Supplement: S3 Table — (DOC) [file pone.0211426.s004.doc]

**Table S3.** **Gene specific primers used for DNA sequencing.**

| **Primer name** | **Primer sequence (5’→3’)** |
| --- | --- |
| CaPPZ3L | TAAAGCAGCACTATCTAAAGG |
| CaPPZ3U | CAGCAACTTCAGAAAATCC |
| CaPPZ4L | GAGTGACATTGGCACATTC |
| CaPPZ4Umod | ATGTGCCAATGTCACCAG |
| CaPPZ5L | GATGACTTGTGAGATGTTG |
| CaPPZ5U | ATCCTTTAGATAGTGCTGC |
| CaPPZ6L | GGACCTTCTCAGTCTTATTG |
| ScPromSacI | GCGAGCTCGTCCTCCAATTCAAC |
| ScPromXbaI | CGTCTAGAAGGAAAGATAAGCAGAG |
