# Supplementary figures and images for: Dissection of the regulatory role for the N-terminal domain in Candida albicans protein phosphatase Z1
Source: PLoS One. 2019 Feb 1;14(2):e0211426. doi: 10.1371/journal.pone.0211426 (PMC6358084; doi:10.1371/journal.pone.0211426)

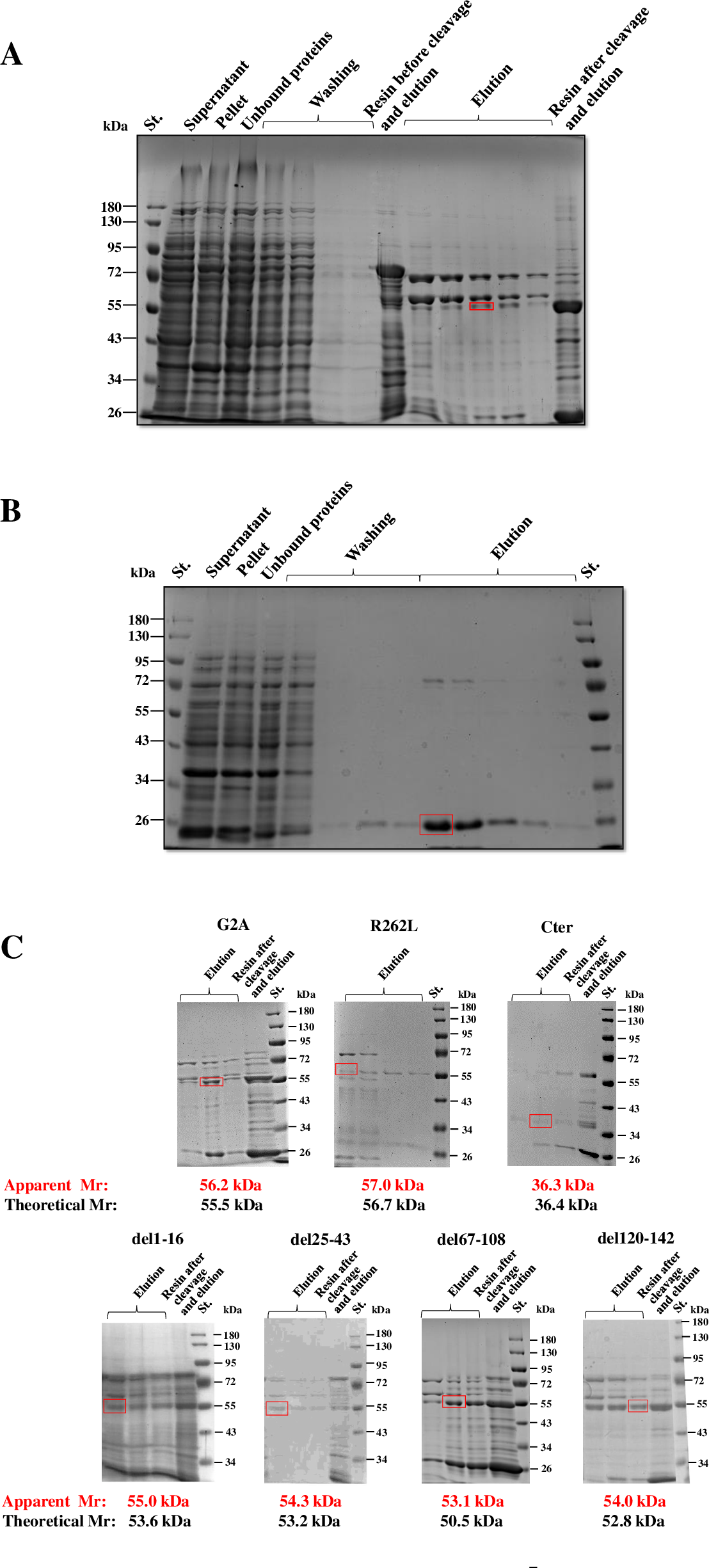

Supplement: S1 Fig — A. Purification of recombinant wild type CaPpz1. Bacterial cell extract was separated to supernatant and pellet by centrifugation and the GST-tagged CaPpz1 was purified from the supernatant on Glutathione Sepharose in batch mode. The bulk of bacterial proteins which did not bind to the resin (unbound proteins) were removed by centrifugation. Weakly bonded proteins were eliminated by washing in six steps. The first, second, fifth, and sixth wash fractions were tested in the gel. The resin with bonded GST-tagged protein was treated with Prescission protease. Fractions containing the cleaved off CaPpz1protein were eluted with five portions of the protease buffer. The third fraction (boxed) was used for further assays. The efficiency of the cleavage was demonstrated by the analysis of an aliquot of Glutathione Sepharose resin before and after protease treatment and elution. The SDS-PAGE analysis of a representative preparation is shown. St. indicates PageRuler Pre-stained Protein Ladder that was used for the estimation of the apparent molecular mass (Mr) of selected bands. The apparent Mr of CaPpz1 is 58.9 kDa that is somewhat more than the theoretical 55.5 kDa value. B. Purification of the N-terminal domain (Nter) of CaPpz1. His-tagged Nter was purified on Ni-NTA-agarose. The supernatant and pellet resulting from centrifugation of the cell extract, unbound proteins, as well as the wash and eluted fractions were analyzed by SDS-PAGE as in S1 Fig. The apparent Mr of Nter is 20 kDa in agreement with the theoretical value of 20.9 kDa. The boxed fraction was used for further assays. C. Testing the purity of mutant CaPpz1 proteins. Recombinant proteins G2A, Cter, del1-16, del25-43, del67-108, and del120-142 were purified on Glutathione Sepharose as the wild type CaPpz1 phosphatase (panel A). Three eluted fractions and the residual resin after cleavage and elution were subjected to SDS-PAGE analysis. The boxed fractions were used for phosphatase assays. The R262L mutant prote [file pone.0211426.s005.tif]

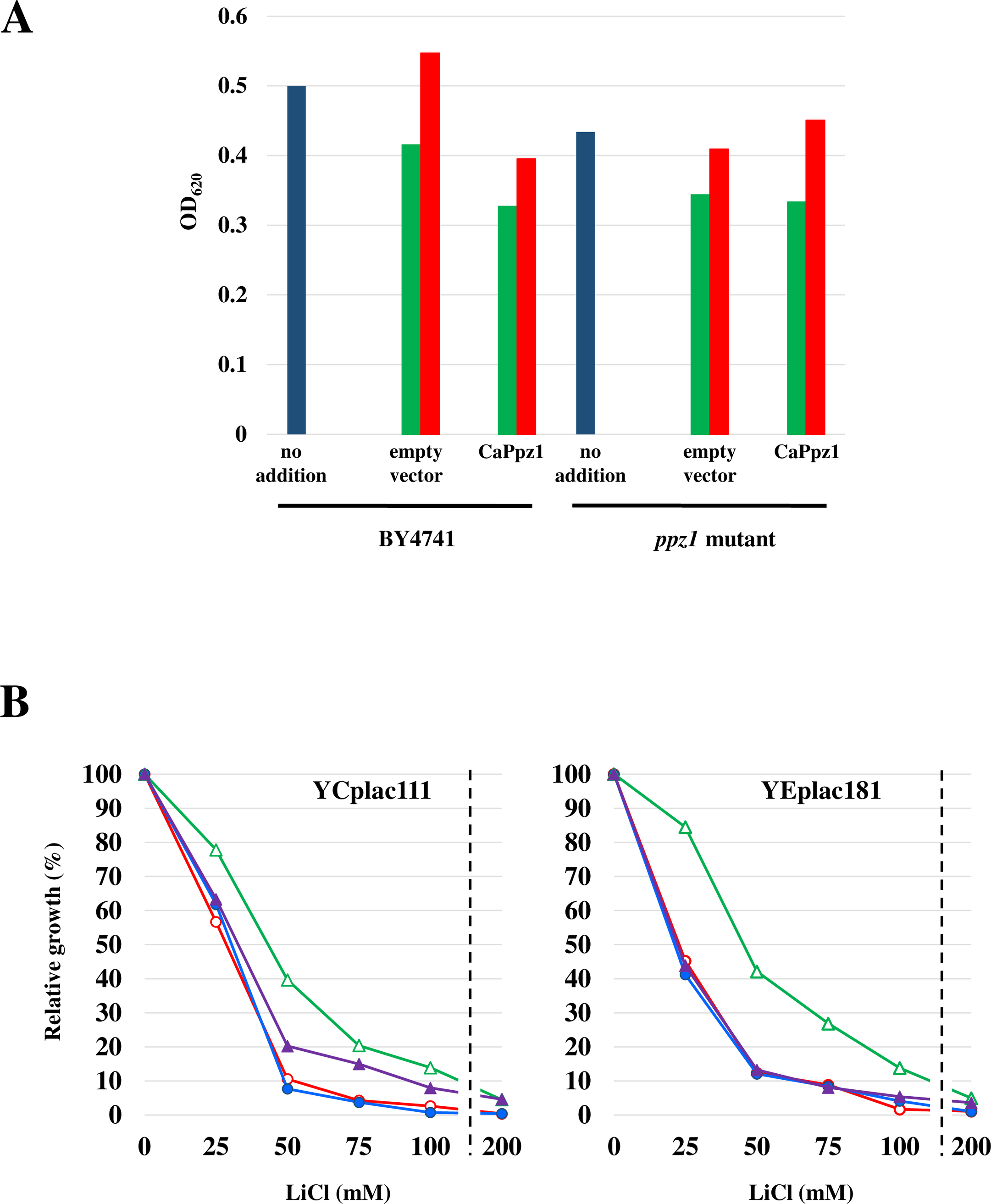

Supplement: S2 Fig — A. Wild type BY4741 and ppz1 deletion mutant S. cerevisiae cells were cultivated without any addition (blue bars), and after transformation with empty or CaPpz1 coding YCplac111 (green) and YCplac181 (red) plasmids. The turbidity (OD620) of triplicate cultures was determined after 19 h incubation. The means of two independent experiments is shown. B. The ppz1 mutant (triangles) and BY4741 control (circles) yeast strains were transformed either with empty (empty symbols) or with CaPpz1 harboring (full symbols) plasmids and were cultivated in the presence of increasing concentrations of LiCl. After 19 h of incubation the optical density of triplicate cultures was measures at 620 nm. The relative growth of the samples cultivated in the absence of LiCl was taken as 100%. The means of two independent experiments is depicted. (TIF) [file pone.0211426.s006.tif]

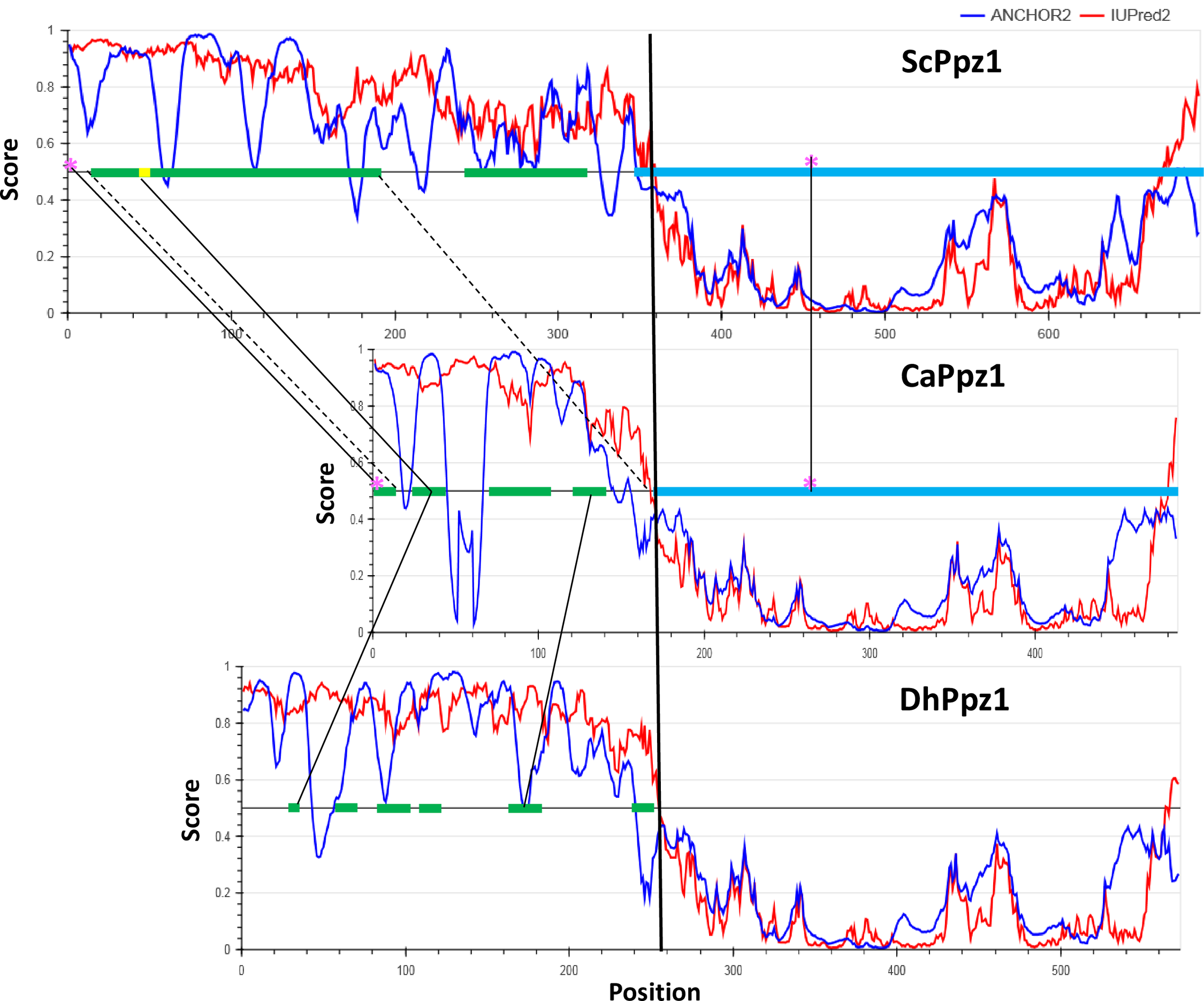

Supplement: S4 Fig — Protein sequences shown in S3 Fig were analyzed by the IUPred2 and ANCHOR2 combined web interface (https://iupred2a.elte.hu/) for protein disorder (red lines) and protein binding regions (blue lines). The graphs were aligned along the conserved catalytic domains. Green bars and a yellow insert indicate deletions, pink stars show point mutations, and blue bars delimit the C-terminal domains as in panel A. Full black lines connect structurally related elements and dashed black lines connect the borders of related regions. (TIF) [file pone.0211426.s008.tif]
