# Supplementary material for: Dissection of the regulatory role for the N-terminal domain in Candida albicans protein phosphatase Z1
Source: PLoS One. 2019 Feb 1;14(2):e0211426. doi: 10.1371/journal.pone.0211426 (PMC6358084; doi:10.1371/journal.pone.0211426)
Supplement: S3 Fig — The amino acid sequences that were investigated by Clotet et al., 1996, Minhas et al., 2012, and in the present work were aligned by the Clustal Omega multiple sequence alignment tool (https://www.ebi.ac.uk/Tools/msa/clustalo/). Green background indicates deletions and pink highlights point mutations. Yellow insert marks the deletion in ScPpz1 that was studied by Minhas et al, 2012. The light blue line above the sequences labels the C-terminal domain of CaPpz1, dark blue extensions mark the limits of the same domain in ScPpz1. Stars under the sequences indicate amino acid residue identities, spot show similarities. (PDF) [file pone.0211426.s007.pdf]

```
ScPpz1      MGNSSSKSSKKDSHSNSSSRNPRPQVSRTETSHSVKSAKSNKSSRSRRSLPSSSTTNTNS   60
CaPpz1      MGSNSSKSA--------------PTLQRSDTNNSTRSTRSNRSIRSRRSISSIKDSQQQ-   45
DhPpz1      MGNNSSKPKD----------LRGSPLSRTDTGNSAK---SSRSIRSRISMSRDDNSTSG-   46
            **..***                  :.*::*.:*.:   *.:* *** *:   .  :

ScPpz1      NVPDPSTPSKPNL------EVNHQRHSSHTNRYHFPSS--SHSHSNSQNELLTTPSSSST   112
CaPpz1      ---Q-----------------------------------------------SDSQQQ--Q   53
DhPpz1      ---SPKTGSKATSRKNSSTSLNNMNSSSHSMNIPINGNNSAHSNNNSSANLSNSFSNLSL   103
               .                                                 : ..

ScPpz1      KRPSTSRRSSYNTKAAADLPPSMIQMEPKSPILKTNNSSTHVSKHKSSYSSTYYENALTD   172
CaPpz1      QQEEEEQSQSQSQQRPQPILSRNNSATSENPILIRRNTNETLQTPNFSD------T----   103
DhPpz1      HKNPSNQHSNNNFNLPP----SMIQVEPKEPILIRRNTGPDGEDTPLSPKNHSFNS----   155
            ::   .: .. . :          .   :.***  .*:.   .    *       .

ScPpz1      DDNDDKDNDISHTKRFSRSSNSRPSSIRSGSVSRRKSDVTHEEPNNGSYSSNNQENYLVQ   232
CaPpz1      --------LSPHS--HSQQQQQQPGSPLST---------------------SMNGNTISR   132
DhPpz1      --------NSPHL--ASSFDNSNPS--------------------------SNPSNNISR   179
                       *    *  .:..*.                          .   * : :

ScPpz1      ALTRSNSHASSLHSRKSSFGSDGNTAYSTPLNSPGLSKLTDHSGEYFTSNSTSSLNHHSS   292
CaPpz1      TST--------NHSI----ISTT-------------------------------------   143
DhPpz1      TST--------NHSH----HSGS--INNYPLSPQP----------------TSSSANGST   209
            : *         **      *

ScPpz1      RDIYPSKHISNDDDIENSSQLSNIHASMENVNDKNNNITDSKKDPNEEFNDIMQSSGNKN   352
CaPpz1      -------------SHKSSSQ---------Q------------------SNLPLKKETTTS   163
DhPpz1      KDL------QEDTSKNFDSQ---------SLNE--------NRNKDVSINNLSHSSTTES   246
                         . : .**         .                   *   :.. . .

ScPpz1      APKKFKKPIDIDETIQKLLDAGYAAKRTKNVCLKNNEILQICIKAREIFLSQPSLLELSP   412
CaPpz1      SLSTNSNTIDIDSLIDKLLNAGFSGKRTKNVCLKNTEIELICASAREIFLSQPSLLELAP   223
DhPpz1      NESDDSGSIDIENLIQRLLDAGYSGKKTKSVCLKNHEIQLICAKARNILLSQPSLLELSP   306
              .  .  ***:. *::**:**::.*:**.***** **  ** .**:*:*********:*

ScPpz1      PVKIVGDVHGQYGDLLRLFTKCGFPPSSNYLFLGDYVDRGKQSLETILLLFCYKIKYPEN   472
CaPpz1      PVKVVGDVHGQYHDLIRIFSKCGFPPKTNYLFLGDYVDRGKQSLETILLLLCYKIKYPEN   283
DhPpz1      PVKVVGDVHGQYGDLIRIFTKCGFPPQTNYLFLGDYVDRGKQSLETILLLLCYKIKYPEN   366
            ***:******** **:*:*:******.:**********************:*********

ScPpz1      FFLLRGNHECANVTRVYGFYDECKRRCNIKIWKTFIDTFNTLPLAAIVAGKIFCVHGGLS   532
CaPpz1      FFLLRGNHECANVTRVYGFYDECKRRCNIKTWKLFIDTFNTLPIAAIVAGKIFCVHGGLS   343
DhPpz1      FFLLRGNHECANVTRVYGFYDECKRRCNIKTWKLFIDTFNTLPIAAIVAGKIFCVHGGLS   426
            ****************************** ** *********:****************

ScPpz1      PVLNSMDEIRHVVRPTDVPDFGLINDLLWSDPTDSPNEWEDNERGVSYCYNKVAINKFLN   592
CaPpz1      PVLNSMDEIRNIARPTDVPDFGLLNDLLWSDPADTINEWEDNERGVSYVFSKVAINKFLS   403
DhPpz1      PVLNSMEEIRNIARPTDVPDFGLLNDLLWSDPADTMNEWEDNERGVSYVFSRVAINKFLQ   486
            ******:***::.**********:********:*: ************ :.:*******.

ScPpz1      KFGFDLVCRAHMVVEDGYEFFNDRSLVTVFSAPNYCGEFDNWGAVMSVSEGLLCSFELLD   652
CaPpz1      KFNFDLVCRAHMVVEDGYEFFNDRTLVTVFSAPNYCGEFDNWGAVMGVSEDLLCSFELLD   463
DhPpz1      KFGFDLVCRAHMVVEDGYEFFNDRTLVTVFSAPNYCGEFDNWGAVMSVSDELLCSFELLD   546
            **.*********************:*********************.**: *********

ScPpz1      PLDSAALKQVMKKGRQERKLANQQQQMMETSITNDNESQQ 692
CaPpz1      PLDSAALKQVMKKEKQERKKST------------------ 485
DhPpz1      PLDSIALKQVMKKGKEERKNAQLQQS-------------- 572
            **** ******** ::*** :
```
